# Unipolar morphology–guided critical isthmus emphasis in a patient with scar-related ventricular tachycardia

**DOI:** 10.1016/j.hrcr.2024.06.013

**Published:** 2024-06-21

**Authors:** Naoya Kataoka, Teruhiko Imamura, Keisuke Uchida, Takahisa Koi, Koichiro Kinugawa

**Affiliations:** Second Department of Internal Medicine, University of Toyama, Toyama, Japan

**Keywords:** Ventricular tachycardia, Activation-recovery interval, Unipolar potential, High-pass filter, Reentry arrhythmias

**Key Teaching Points**

• Conventional endocardial substrate mapping techniques may occasionally fail to detect the critical isthmus of scar-related reentrant ventricular tachycardias, particularly in instances where the critical isthmus is situated within intramural or epicardial layers.
• The activation-recovery intervals (ARIs) in the injured area owing to ischemia have been observed to be shorter compared to those in healthy myocardium.
• Our proposed mapping technique, “ARI mapping,” detected critical isthmuses by employing a 0.05 Hz high-pass filter for unipolar potentials, positioning a reference electrode in the inferior vena cava, and automatically detecting maximum dV/dt.

## Introduction

Scar-related reentrant tachycardia constitutes a primary etiology of monomorphic ventricular tachycardias (VT) in patients with underlying cardiac pathologies. In the ventricle, the circuit is 3-dimensionally composed, involving the endocardium, the midlayer, and the epicardium.^1^ However, currently proposed mapping techniques for detecting VT isthmus focus on bipolar potentials, such as isochronal late activation mapping, fractionation mapping, or bipolar voltage mapping, all of which reflect near-field electrophysiological activities but unfortunately lack deeper layer information.^2^^,^^3^ In terms of unipolar electrocardiograms, voltage mapping is currently the only methodology for evaluating intramural or epicardial conduction abnormalities.^4^

Recent literatures have demonstrated that local unipolar electrocardiograms show heterogeneity within the ventricle, with shorter activation-recovery intervals (ARIs) observed in the VT isthmus compared to those in other areas.^5^^,^^6^ However, the primary limitation of these studies was the high-pass filter set at 2 Hz in the nominal setting of the 3-dimensional mapping system, which critically affects ST-T morphology, leading to an erroneous assessment of ARIs.^7^

In the evaluation of ARIs using a high-pass filter set at 0.05 Hz in a case of scar-related VT associated with ischemic cardiomyopathy, we hypothesized that if unipolar potentials reflect far-field electrophysiological characteristics, their morphologies would be influenced by intramural or epicardial electrophygiological characteristics, which unquestionably occurs in the VT isthmus, thereby impacting on ARIs and facilitating automatic visualization of the VT isthmus.^8^

## Case report

### Medical history

A 47-year-old male patient was emergently admitted owing to cardiogenic shock, ascribed to recurrent polymorphic and monomorphic sustained VTs refractory to medical therapies including amiodarone, lidocaine, and landiolol, concurrent with ischemic cardiomyopathy characterized by a left ventricular ejection fraction of 15%. Coronary angiography revealed total occlusion of the left anterior descending artery with collateral flow from other branches, and 75% stenosis in both the right coronary artery and the left circumflex artery. Despite urgent catheter ablation performed owing to persistent VTs following percutaneous coronary artery interventions for the left anterior descending artery, it failed to terminate the persistent VTs.

Following the implantation of the percutaneous left ventricular assist device, the Impella 5.5 (Abiomed, Danvers, MA), he was transferred to our hospital for receiving more intensive therapies together with heart transplant listing. Despite the clinical decision of device upgrade to a durable left ventricular assist device, the patient had persistent electrical storm. We decided to perform catheter ablation again to stabilize hemodynamics before durable left ventricular assist device implantation.

### Patient characteristics

**Figure 1:**
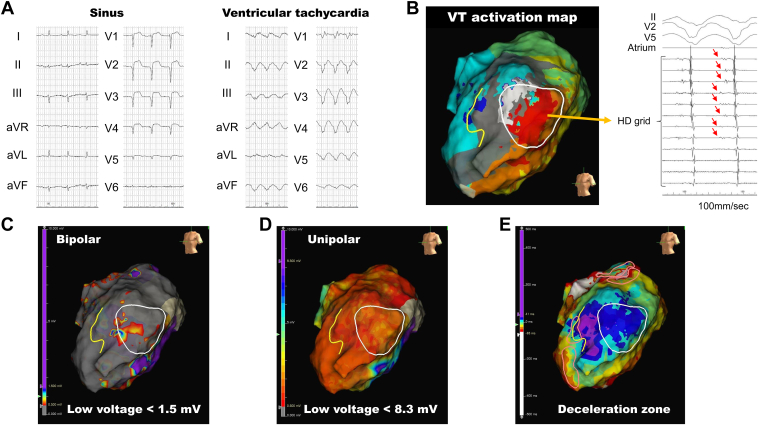
The targeted ventricular tachycardia (VT) and the endocardial mapping. **A:** The left panel shows the electrocardiogram during sinus rhythm; the right panel, VT. **B:** Activation map of the targeted VT. The white circled area indicates the location where the diastolic potentials were recorded during the tachycardia. **C:** Bipolar voltage map during sinus rhythm, with a cut-off for low voltage set at <1.5 mV. **D:** Unipolar voltage map during sinus rhythm, with a cut-off for low voltage set at <8.3 mV. **E:** Isochronal late activation map for revealing the deceleration zone, which is depicted by the pink circles. The yellow lines indicate localized functional lines of block detected by the isochronal activation map.

The echocardiographic testing revealed akinesis in the left ventricular septum and anterior wall, and dyskinesis in the apex, with a left ventricular end-diastolic dimension of 74 mm. During sinus rhythm, the electrocardiogram displayed a QS pattern in leads V_1_–V_5_, accompanied by horizontal ST-segment elevation (left panel in Figure 1A). Clinical monomorphic VT demonstrated a northwest axis and a right bundle branch block pattern (right panel in Figure 1A).

### Catheter ablation

The catheter ablation procedure using the 3-dimensional mapping system EnSite X (Abbott Laboratories, Abbott Park, IL) was conducted under deep sedation using intravenous infusion of propofol and dexmedetomidine, with the use of a laryngeal mask. Left ventricular endocardial substrate mapping using an HD Grid mapping catheter (Abbott) was performed during sinus rhythm, recording local unipolar electrograms with a reference electrode positioned in the inferior vena cava, as previously reported.^9^ Afterward, programmed stimulation was conducted to induce monomorphic VTs. The induced VT, identical to the clinical presentation (Figure 1A), was mapped under relatively stabilized hemodynamics maintaining a mean blood pressure exceeding 60 mm Hg owing to the Impella 5.5 and intravenous noradrenaline infusion.

Diastolic potentials during the tachycardia were recorded in the lateral wall, as indicated by the white circled area in Figure 1B. Concealed entrainment was achieved through continuous pacing in the area, with the postpacing interval equaling the tachy-cycle length plus 20 ms. Although radiofrequency ablation at 30 watts for 30–60 seconds was performed in this region, only transient termination of the tachycardia was achieved, and it immediately recurred upon programmed stimulation. Hence, the critical isthmus was deemed to be located within the intramural or epicardial regions, instead of the endocardium. Owing to the prolonged duration of the procedure, we decided to schedule epicardial ablation in a subsequent session, and the current session was concluded.

### The procedure review

While the present VT could be fortunately mappable under stabilized hemodynamics with mechanical circulatory support, the majority of VT cases generally cannot undergo activation mapping during tachycardias owing to hemodynamic instability. Hence, the procedure was reviewed for detecting the critical isthmus, which encompassed not the endocardium but the intramural or epicardial layers, utilizing endocardial substrate mapping during sinus rhythm.

#### Conventional mappings

Initially, voltage maps using both bipolar and unipolar potentials were evaluated. The bipolar voltage map, with a cut-off for low voltage set at <1.5 mV, revealed a broadly scarred area indicated in gray and patchy low-voltage zones indicated in red within the diastolic potential recording area during VT (Figure 1C). The unipolar voltage map, with a cut-off for low voltage set at <8.3 mV, also demonstrated a broadly low-voltage area matching with those of the bipolar voltage (Figure 1D). However, these voltage maps could not reveal the critical isthmus, represented by the white circle in Figure 1, including the unipolar map, which is believed to reflect epicardial potentials. The deceleration zone (>3 isochrones within 1 cm radius), identified by pink circles on the isochronal late activation map, was located at the entrance of the VT isthmus, coinciding with the area marked by the yellow line indicating functional lines of the block (Figure 1E). However, it did not align with the diastolic potential recording area during VT (Figure 1E).

#### ARI mapping

**Figure 2:**
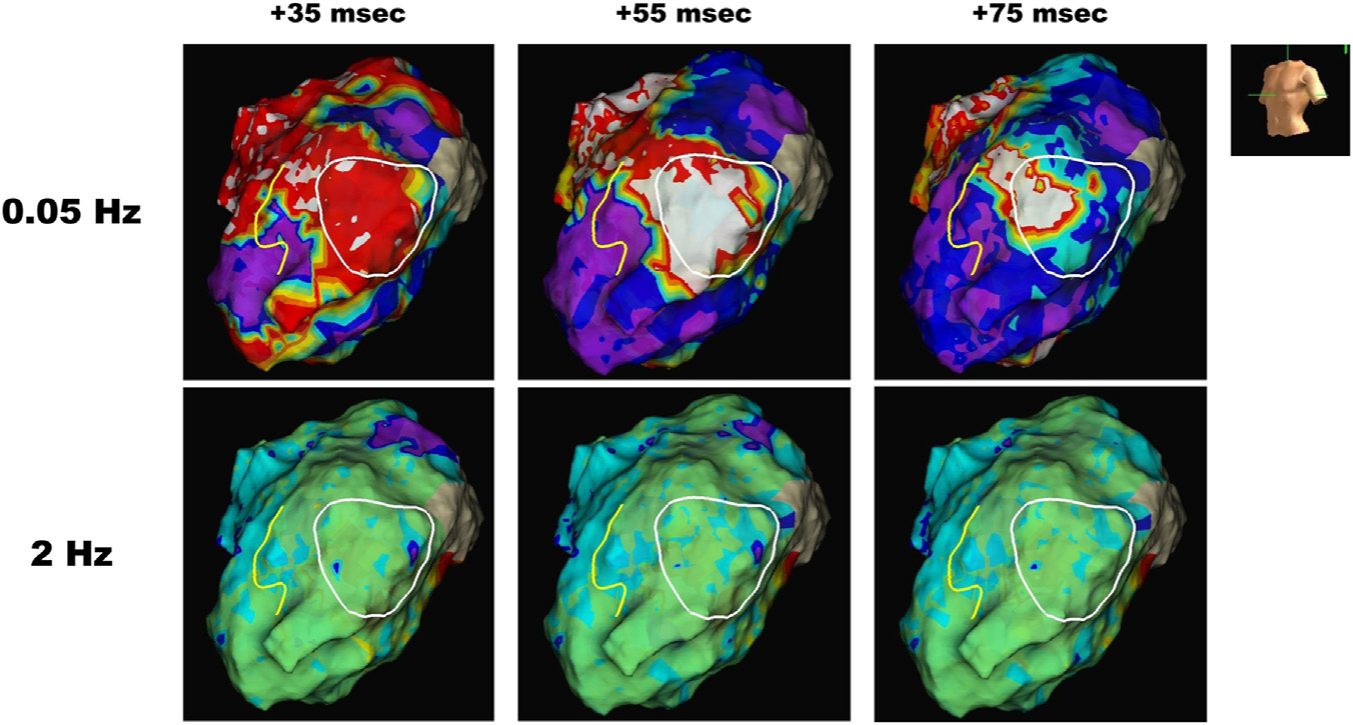
Unipolar activation maps with 2 high-pass filter settings. Figures where the duration from the onset to the maximum dV/dt point of the unipolar waveform was color-coded with reference to the S wave of lead I in surface electrocardiogram. The number of milliseconds indicates the blanking time from the S wave of lead I.

Unipolar maps using local morphologies were reconstructed using the Auto Outlier Filtering function of EnSite X, which automatically removes outliers. The duration from the S wave in lead I, easily identifiable as an annotation, to the maximum dV/dt point automatically identified by the EnSite system, was evaluated. We designated the proposed new method as “ARI mapping.” For QRS component filtering, the blanking time from the S wave in lead I was dynamically adjusted from 35 to 75 ms per 20 ms (Figure 2). The nominal setting of the unipolar high-pass filter at 2 Hz could not visualize the critical isthmus at any adjusted time (the lower line in Figure 2).

Notably, by adjusting the high-pass filter to 0.05 Hz, the critical isthmus was successfully visualized as the short-duration area indicated by the white region, and such a trend remained irrespective of any changes in time adjustment (the upper line in Figure 2).

**Figure 3:**
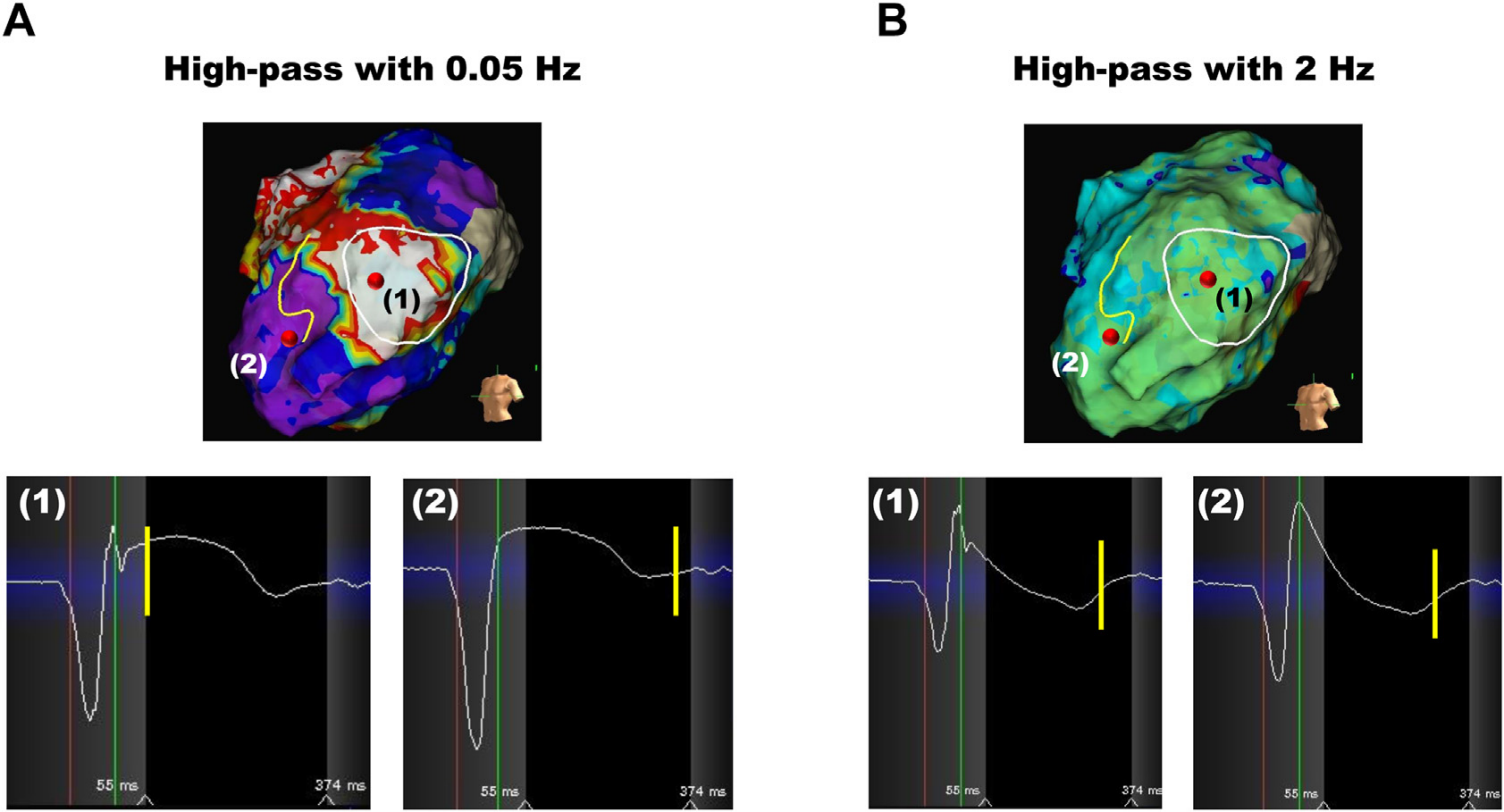
Unipolar potential morphologies. Maps identifying from the S wave in lead I to the maximum dV/dt point using the local unipolar morphologies. (1) and (2) indicate the critical isthmus point and another area, respectively, which are the same points irrespective of the filter setting. **A:** High-pass filter set at 0.05 Hz. **B:** High-pass filter set at 2 Hz.

When examining the local unipolar potentials, it was observed that at 0.05 Hz the J-ST segment exhibited a dome-shaped elevation, while at 2 Hz it consistently demonstrated a steep decline in a down-sloping fashion, accompanied by a negative T wave (Figure 3). In the high-pass filter set at 0.05 Hz, a convex-type J-ST elevation was observed in the VT isthmus, as indicated in (1) in Figure 3, prompting the placement of the maximum dV/dt point (the yellow bars) at the upslope of the T peak (Figure 3A). Conversely, the other area, as indicated in (2) in Figure 3, displayed a horizontal J-ST segment with a shallow negative T wave, resulting in the maximum dV/dt point being set at the upslope of the T wave (Figure 3A). In the high-pass filter set at 2 Hz, the local unipolar morphologies were similar, resulting in no significant difference in the duration from the S wave in lead I to the maximum dV/dt point between the VT isthmus and other areas (Figure 3B).

### Following the procedure

The patient received an implantable cardioverter-defibrillator. Subsequently, he had multiple VT recurrences while awaiting left ventricular assist device implantation, all of which were successfully terminated with antitachycardia pacing therapy. However, he experienced sudden death owing to cardiac arrest following defecation 3 weeks after undergoing catheter ablation, despite receiving continued percutaneous left ventricular assist device support.

## Discussion

The present case-based study identified a novel method for detecting scar-related reentrant VT isthmus through endocardial substrate mapping during sinus rhythm. In this case, where the VT isthmus exists within deep layers, conventional methods of endocardial mapping proved inadequate for identification. Our proposed new method, “ARI mapping,” which uses automatic maximum dV/dt point detection with a high-pass filter set at 0.05 Hz in the unipolar potentials, enabled clear visualization of the VT isthmus, even when it exists within the intramural or epicardial layers. Our case was fortunately able to perform VT activation mapping thanks to the hemodynamic stabilization by the percutaneous left ventricular assist device support; however, owing to the hemodynamic instability of most VT (>70%), substrate mapping during sinus rhythm is required in most procedures.

### Disadvantages of conventional mappings

Endocardial voltage maps using local bipolar or unipolar potentials could readily identify impaired myocardial regions. Bipolar cases reflect near-field potentials, indicating endocardial information, while unipolar cases encompass not only the endocardium but also the intramural or epicardial layers.^10^ These voltage-based mapping methods have a critical limitation: they do not provide data on the wavefront propagation pattern, which is essential for detecting VT isthmus.^11^ Therefore, emerging techniques for detecting VT isthmus using substrate mapping have focused on analyzing the propagation of bipolar potentials, as depicted by the isochronal late activation mapping (Figure 1E).^12^^,^^13^

Given the characteristics of bipolar potentials reflecting near-field information, isochronal late activation mapping sometimes fails to detect VT isthmus, especially in cases where the VT isthmus exists in the intramural or epicardial layers, as in our case. In fact, the site recording diastolic potentials during the VT outlined in white differed from the areas of the deceleration zone outlined in pink (Figure 1E).

### Advantages of ARI mapping

It is well known that left ventricular action potential durations are shortened following myocardial infarction owing to ion channel remodeling, including changes in gap junction or connexin 43 kinetics.^14^ Particularly with respect to potassium channel β-subunits, upregulation is observed in electrical remodeling, leading to an increase in potassium conductance and shortened action potential durations.^15^ Shortened action potential duration, meaning shortened refractory periods, facilitates reentrant circuits; therefore, it has been widely accepted that the VT isthmus demonstrates shorter action potential durations compared to other areas.^6^

Owing to the impossibility of direct measurements of action potential durations in vivo, ARIs, defined as the interval between the times of minimum derivative of the QRS and maximum derivative of the T wave in unipolar electrograms, are proposed as an alternative method for estimating action potential durations.^16^ Several methods for detecting the end of ARIs, particularly in cases with and without negative T waves, have been proposed; however, the Wyatt method is increasingly recognized as the correct approach.^8^ Therefore, the present case adopted the Wyatt method for detecting the end of ARIs and compared them with high-pass filters set at 2 Hz and 0.05 Hz. It is noteworthy that using the 0.05 Hz filter, the VT isthmus could be visualized clearly, whereas this was not the case with the 2 Hz filter, regardless of how much the beginning of the ARI was altered (Figure 2). It was evident that the difference was due to variations in the unipolar potential waveform based on the difference in high-pass filter settings (Figure 3). We acknowledge that this method does not precisely correlate with ARI, as it necessitates the adjustment of the ARI beginning point. Yet, this novel approach, capable of detecting VT isthmus in the myocardial mid-to-deep layers with minimal adjustment to the ARI beginning point, represents a significant advancement not previously attainable.

### Potential limitations of ARI mapping

If the endocardial myocardium remains electrically healthy while only the epicardial tissues form scars, which are sometimes observed in cases of nonischemic etiologies, this novel technique may be misleading because unipolar potentials are affected by both near-field and far-field electrophysiological characteristics. The ARI mapping technique may be particularly useful for identifying areas with shortened ARI within scar tissue. To determine the optimal cases for the ARI mapping technique, further validation study through multiple case studies is warranted.

### Clinical perspective

In cases where the VT isthmus is located within the endocardium, conventional substrate mapping techniques such as isochronal late activation mapping may suffice. However, for cases where the VT isthmus extends into deeper layers, our proposed new method, termed “ARI mapping,” should demonstrate efficacy in detecting the VT isthmus.

## Conclusion

ARI mapping, whose key factors for establishment include setting the high-pass filter of unipolar potentials to 0.05 Hz, positioning a reference electrode in the inferior vena cava, and automatically detecting maximum dV/dt regardless of the presence of negative T waves, was expected to correlate with VT isthmus.

## Disclosures

There were no relationships with industry.
